# Therapeutics

**Published:** 1878-01

**Authors:** 


### IV. Therapeutics.

Quinine Exanthem.—Prof. Köbner, of Breslau (*Berl. Klin. Wochenschr.*, May 28 and June 8), reports a case where a syndrome closely resembling scarlet fever resulted from even a small dose of quinine. The symptoms consisted of a chill, sometimes repeated, a feeling of precordial anxiety, nausea, vomiting, intense headache, high fever and angina, followed, after a few hours, by an erythematous eruption on the face, which spread rapidly over the whole body. This eruption was attended by intense itching and burning, by slight œdema of the face and injection of the conjunctiva. The color disappeared for a moment under pressure. The angina affected only the posterior wall of the pharynx, the soft palate and pillars being normal. The symptoms abated, and desquamation began after a variable length of time, according to the amount of quinine taken. Three times within five months was the patient attacked in this manner.

Dr. Von Hensinger, of Marburg, reports two cases similar to the above, except that the eruption was confined to the face. Both patients were women; one of them had formerly taken quinine without inconvenience. M. E. K.

On Iodoform.—Lailler. (*La France Médicale*, Oct., 20, 1877.) Extract from a Clinical Lecture delivered at the Saint Louis Hospital.

Iodoform is a compound of iodine, hydrogen and carbon, or the hydriodide of carbon, and was discovered by Sérallas in 1822. In 1834, Dumas gave it the name iodoform, because of the analogy it presented to chloroform and bromoform.

It is obtained by the action of an alkali upon an alcoholic solution of iodine, and it is a crystalline body in hexagonal lamellæ, of a brilliant lemon-yellow color, and a peculiar, penetrating and persistent odor, which is readily recognized when it has once been perceived. It is partially volatilized under the action of a moderate heat. While it is scarcely soluble in water, it is readily dissolved in boiling alcohol and ether.

Among the physicians who have carefully studied iodoform is M. Bouchardat. Upon several occasions he has called attention to this product, and has demonstrated that it contains 90 per cent. of iodine. He has likewise discovered a special procedure for obtaining it.

In 1860, M. Righini, of Navarre, of the Brussels Society of Medical and Natural Sciences, read a very complete manuscript upon iodoform, which was written in a rather exaggarated style.

It would seem that in France first iodoform was specially studied and employed in a systematic and sustained manner.

Thus, in 1853, Messieurs Moretin and Humbert presented to the Academy a work on the subject, which was followed by the inaugural thesis of M. Moitre of similar title. But, in spite of all these interesting publications, iodoform was neglected for several years.

In 1859, when I was the physician of the Lourcine Hospital, I began to employ it, and the favorable results obtained, determined me to make a wider application of its value. During my service of three years I had no reason to regret this course. Upon my arrival at the St. Louis Hospital, I had an opportunity to use it still more extensively, and to decide in the clearest manner as to its incontestable value. In order to be assured of this, it is only necessary to read the papers since published by Besnier, Demarquay, Féréol, Maillard, Nieszkowski and Petiteau.

What is the mode in which iodoform should be employed?

Up to the present time, its use as internal medicament has given results of little value, as the researches of M. Righini and myself clearly show. This is a question which has yet to be studied.

Its topical action is very marked in a twofold direction—it is both anæsthetic and promotive of cicatrization.

Its action as an anæsthetic, permits us to make use of it to relieve the pain of fissures of the anus, hæmorrhoids, ulcerations of the throat, ulcerative cancers, and in particular, those of the face, mouth, breast, and cervix uteri. The action of iodoform, in all these cases, is quite rapid. In order to use it to the best advantage, it should be reduced to a very fine powder, and be carefully applied to every part of the diseased surface. The simplest method of obtaining it in this form, is to dissolve it in sulphuric ether, and then permit the ether to evaporate, when it is left in the form of an impalpable powder. In certain regions, particularly that of the anus, it is well to associate it with other substances, cacao butter for example, as the suppositories thus made, facilitate the application of the remedy. In general, the employment of the powder only is preferable, and I will add, in this connection, that you may employ it fearlessly in considerable quantity, as I have never seen accidents from such a course. I have never, it is true, made use of very large amounts at one time. But Demarquay, who has employed it in the treatment of a great number of wounds, *largâ manu*, has never had occasion to regret it.

The action of iodoform, by which it favors cicatrization, is quite as remarkable as its anæsthetic property. In general terms, it may be said that the substance modifies ulcers of every variety. It is often astonishing to note the rapidity with which, under its influence, favorable changes and cicatrization occur, in soft chancres, ulcerative buboes, mucous patches, and syphilitic ulcerations of all kinds. Phagedena is often arrested in its course by a few applications of powdered iodoform, and not unfrequently onyxis is completely relieved in a few days after its topical use, though the last named affection is well known for its rebellious character and its ordinary duration of several months.

You have seen the results obtained in my service in all the cases just described. For some time past I have attempted, with success, to treat, by the employment of this remedy, scrofulous ulcers, lupus and epithelioma. You can note the subjects of these various disorders in the wards, and the sensible amelioration of their condition which has resulted.

Under the influence of iodoform, inflammatory phenomena disappear, and granulations lose the flabby look which is characteristic of many scrofulous ulcers. In fact, it is often necessary to repress exuberant granulation by the employment of the crayon of nitrate of silver. The wound, thus modified, rapidly cicatrizes, and the progress accomplished in the space of one day only is often remarkable.

How does iodoform operate to produce these results? M. Féréol admits that, as a powder, it is inert; for myself, I believe that the iodine is the effective element, for, as has been said, the powder contains ninety per cent. of the latter.

I have hitherto referred merely to the value of the medicament. It remains for me to indicate the objections to its use. Iodoform has a most penetrating and insupportable odor, on account of which it is often necessary to abandon its employment, and, for the same reason, patients sometimes absolutely refuse the treatment.

It would be well to dispose of this objection. Renaut, concurring with me in the belief that iodine is the active principle, concluded to make a compound of talc and iodine, which he termed "iodated talc," and with which various experiments were made by him in my wards. The results were fairly satisfactory, but much less perfect than those due to iodoform itself. It has also been thought that camphor in powder would serve as a substitute, but comparative trials have not been favorable to the use of the latter. Its odor, truly, is not disagreeable; but then it presents none of the properties of iodoform, does not modify the character of ulceration, nor act as an anæsthetic.

I have found nothing to replace iodoform, the latter having, in my judgment, more valuable qualities than inconveniences. Certain precautions are to be observed in its application. It should never be applied to other than perfectly clean wounds. Atomization of warm water will answer for the purpose of cleansing them; then they should be carefully dried, and the powder applied. The fineness of the powder permits of its entrance in all the anfractuosities of the ulcer. Once applied, Féréol advises to cover the wound with charpie, oakum, or diachylon. The dressing may be repeated daily, or twice daily, at the outset — progressively less frequent as cicatrization advances.

In order to better apply the iodoform to deep lesions, situated, for example, in the throat or uterine neck, I have devised a procedure, which I have experimented with for several days, and which has thus far given good results. It consists in dissolving one part of iodoform in ten of ether, and atomizing the solution with Richardson's apparatus.

By this means, the atomizing tube being curved, and of sufficient length, the jet of the spray can be easily directed upon the parts mentioned, and all the diseased portions at once covered with a more or less thick layer (according to the requirements of each case), of a fine and impalpable powder, which adheres to the surface much more intimately and permanently than by other methods.

To give you an idea of the extent to which the employment of iodoform has increased, I append some figures which indicate the annual consumption of this product in the hospitals of Paris : In 1859, 250 grammes were used ; in 1866, 600 grammes ; in 1869, after the publication of the researches of Demarquay, Besnier, and Féréol, 20 kilogrammes ; in 1873, the time of the siege, 33 kilogrammes ; lastly, in 1875, 28 kilogrammes (about 56 pounds).

So you see this remedy has made its own way. It will remain in the arsenal of therapeutics, and render incontestable service when judiciously employed.

Urethritis Provoked by Arsenical Preparations.—*Jour. de Médecine*, Nov. 1877.) M. Saint-Philippe describes, in the *Gazette Médicale de Bordeaux*, certain accidents to the urethra from the use of arsenical preparations. A patient of M. Saint-Phillippe, suffering with malarial fever, was given arsenic, of which he took only four milligrammes. At the end of some days, however, he was seized with symptoms of poisoning, vomiting, colic, diarrhœa, etc., and was obliged to suspend treatment. The day after he was attacked with urethritis, which was treated in the classical way, and lasted fifteen days. The patient declared that he had not been exposed by any veneraal act for more than two months.

Another patient, suffering with scaly affection of the skin, was given arsenic, but, by mistake, took double the dose prescribed, so that in eight days he had taken sixteen milligrammes of the medicine. The same accidents followed, the urethritis being treated as usual, and lasting about fifteen days. In this case, also, the patient had not been exposed by venereal acts. M. Saint-Phillippe believes, then, that it is rational to suppose that the arsenic, in being eliminated by the urinary organs, may produce the same accidents which it does in the digestive system. He believes the irritation to be toxic, rather than physiological, which accounts for its rarity in practice.

L. W. C.

FORMULA FOR NIGHT SWEATS.—Porcher. (*L'Union Médicale*, Nov. 6, 1877.)

| | | |
|---|---|---|
| Sulphate of Atropine - - - | 1 | centigramme. |
| Extract of Gentian - - - | 10 | " |

Acacia q. s. to make 10 pills.

Dose, one or two a day for the night sweats of consumptives.

ANTI-ASTHMATIC ELIXIR.—Desnos. (*L'Union Médicale*, Nov. 10, 1877.)

| | | |
|---|---|---|
| Snakeroot - - - - . | 3 | grammes. |
| Water - - - - - - | 125 | " |

Boil until reduced one half, strain and add iodide of potassium, 6 grammes.

After cooling, add

| | | |
|---|---|---|
| Brandy - - - - - - | 60 | grammes. |
| Syrup of Opium - - - - | 120 | " |

Filter. Dose two or three tablespoonsfull a day for asthmatics; the first before breakfast and the other between meals.

